# Supplementary material for: Vertical redistribution of principle water masses on the Northeast Greenland Shelf
Source: Nat Commun. 2022 Dec 10;13:7660. doi: 10.1038/s41467-022-35413-z (PMC9741604; doi:10.1038/s41467-022-35413-z)
Supplement: Supplementary file 1 — Supplementary Information [file 41467_2022_35413_MOESM1_ESM.pdf]

Supplementary Information for

Vertical redistribution of principle water masses on the Northeast Greenland Shelf

Caroline V. B. Gjelstrup*, Mikael K. Sejr, Laura de Steur, Jørgen Schou Christiansen, Mats A. Granskog, Boris P. Koch, Eva Friis Møller, Mie H. S. Winding, & Colin A. Stedmon

*Corresponding author: Caroline V. B. Gjelstrup

Email: cvbgj@aqua.dtu.dk

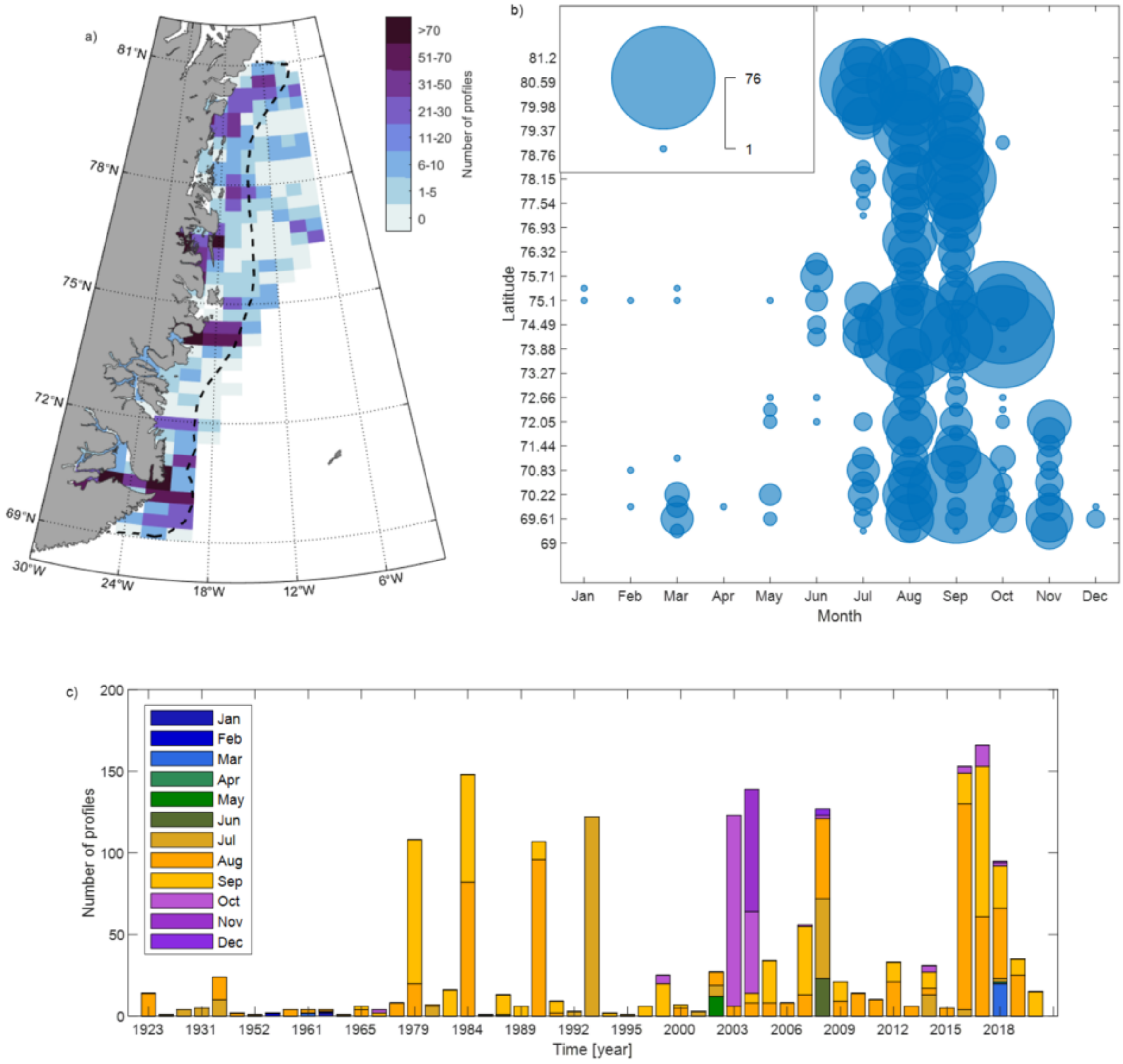

Fig. S1. Spatial and temporal distribution of the hydrographic data set complied for the current study. a) Total number of profiles per 1° longitude by 1/3° latitude bin. The black dashed line indicates the near-shore shelf region. b) Profile density for each month per 1/3° latitude bin within the near-shore shelf region. The number of profiles is given by the area of the bubble. c) Number of profiles per year colored by month within the near-shore shelf region.

Supplementary note 1. Spatial variability of temperature and salinity within the fjords was evaluated by calculating semivariance as a function of the geographical distance separating a pair of observations. Observations close in space are expected to be more similar (lower semivariance) than those separated by large distances. Calculating the semivariance for all possible pairs of observations and separation distances allows us to assess the spatial continuity of temperature and salinity and thus gives an indication of the extent of spatial variability within each fjord. Semivariance, γ_x, as a function of separation distance h associated with variable x is calculated as follows:

$$\gamma_x(h) = \frac{1}{2N(h)} \sum_{i=1}^{N(h)} \left(Z(u_i) - Z(u_i + h)\right)^2 \tag{S1}$$

Where, N is the number of data pairs for a given separation distance and Z is the numeric value of the observation at the location given by u_i. A separation distance increment of 5 km was chosen for each iteration of the computation. Due to non-uniformly spaced data, a distance tolerance of half the separation distance increment was applied.

Equation (S1) was evaluated based on 1 m binned profiles and only calculated between observations collected in the same year as not to alias temporal with spatial variability. The semivariance was then averaged across years to arrive at the final estimate. All data pairs regardless of direction were included. The resulting statistic is therefore independent of direction.

Semivariances were generally small for depth levels greater than 30 m, and no systematic relationships between semivariance and separation distance were found (Supplementary Figure 2). Surface waters showed a higher degree of variability, but was neither related to separation distance. An exception was seen for the 15 m depth level in Dijmphna Sound where semivariance for both temperature and salinity increased as the distance between the observations increased (Supplementary Figure 2 b&c). The overall small and spatially unstructured semivariances give confidence that temporal trends in fjord properties are not an artefact of spatial variability.

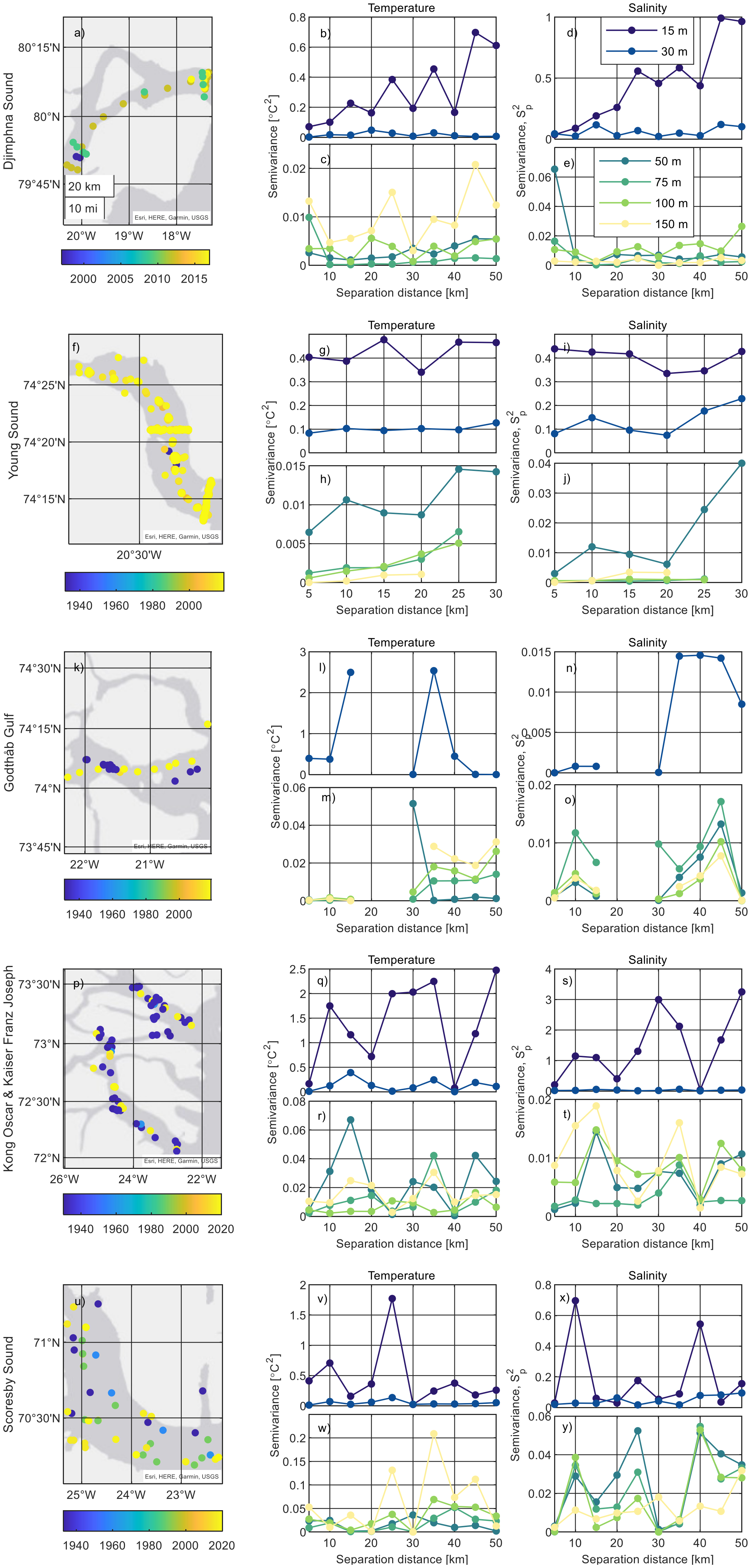
Djimphna Sound
a)
80°15'N
80°N
79°45'N
20 km
10 mi
Esri, HERE, Garmin, USGS
20°W
19°W
18°W
2000
2005
2010
2015
Temperature
Salinity
b)
c)
d)
e)
15 m
30 m
50 m
75 m
100 m
150 m
Semivariance [°C^2]
Semivariance, S_p^2
Separation distance [km]
Young Sound
f)
74°25'N
74°20'N
74°15'N
20°30'W
1940
1960
1980
2000
g)
h)
i)
j)
Godthåb Gulf
k)
74°30'N
74°15'N
74°N
73°45'N
22°W
21°W
l)
m)
n)
o)
Kong Oscar & Kaiser Franz Joseph
p)
73°30'N
73°N
72°30'N
72°N
26°W
24°W
22°W
2020
q)
r)
s)
t)
Scoresby Sound
u)
71°N
70°30'N
25°W
24°W
23°W
v)
w)
x)
y)

Fig. S2. Spatial variability of fjord properties. Spatial distribution of observations by year (left column) and semivariograms for temperature (middle column) and salinity (right column) at the 15 m, 30 m, 50 m, 75 m, 100 m and 150 m depth levels for Dijmphna Sound (a-e), Young Sound(f-j), Godthåb Gulf (k-o), Kong Oscar and Kaiser Franz Joseph fjord (p-t) and Scoresby Sound (u-y). Panels a, f, k, p & u were prepared with the Mapping Toolbox for MATLAB version 5.1.

Supplementary note 2. Variance in the hydrographic metrics used throughout the study was investigated with the objective of comparing the variance arising due to sampling location and the variance arising due to temporal change in water mass properties. This was done for the repeat shelf transect outside Young Sound and the shelf area bounded by [“-16.97”, “78.00”, “-13.45”, “79.06”] sampled as part of the Fram Strait monitoring programme as well as several research cruises. The Young Sound shelf transect is 27 km long and has been sampled every year since 2003. Data from within the second area is available for 1956, 1979, 1984, 1993, 1998, 2000, 2002, 2012, 2016-2019. The variance of each metric is calculated for each year. Taking the average of these variances, gives a robust estimate of the variance arising due to differences in sampling location within the two areas. This value is compared with the total variance in each metric, calculated from all available summer samples collected within the two areas, respectively. Supplementary table 2 shows that variance is small when evaluated for a single year compared to when data from all years is included. This is formally corroborated with a two-sided F-test evaluating the null hypothesis, H_0: $\sigma_1^2 = \sigma_2^2$ against the alternative hypothesis, H_A: $\sigma_1^2 \neq \sigma_2^2$. The null hypothesis is rejected in all cases with minimum significance level of 5 %. Thus, the variance arising due to difference in sampling location is statistically different from the variance due to temporal changes in the hydrographic metrics at the two locations investigated.

Table S1. Variance in four hydrographic metrics for the shelf transect outside Young Sound and Fram Strait. Group 1 is the mean of the variance calculated for each individual year and therefore represents the variance arising due to sampling location along the transect. Group 2 represents the total variance in each metric. N gives the number of observations in each group. The F-test statistic for comparison of variances is given in the bottom row.

	N	*PW temperature*	*PW salinity*	*AW temperature*	*AW salinity*
			Young Sound		
Group 1	17	0.0004	0.0078	0.1313	0.0101
Group 2	130	0.0026	0.2723	0.2723	0.0128
F_0		5.87	3.68	1.59	1.27
			Fram Strait		
Group 1	12	0.0002	0.0236	0.0715	0.0038
Group 2	95	0.0031	0.1508	0.2692	0.0064
F_0		13.75	6.39	3.76	1.66

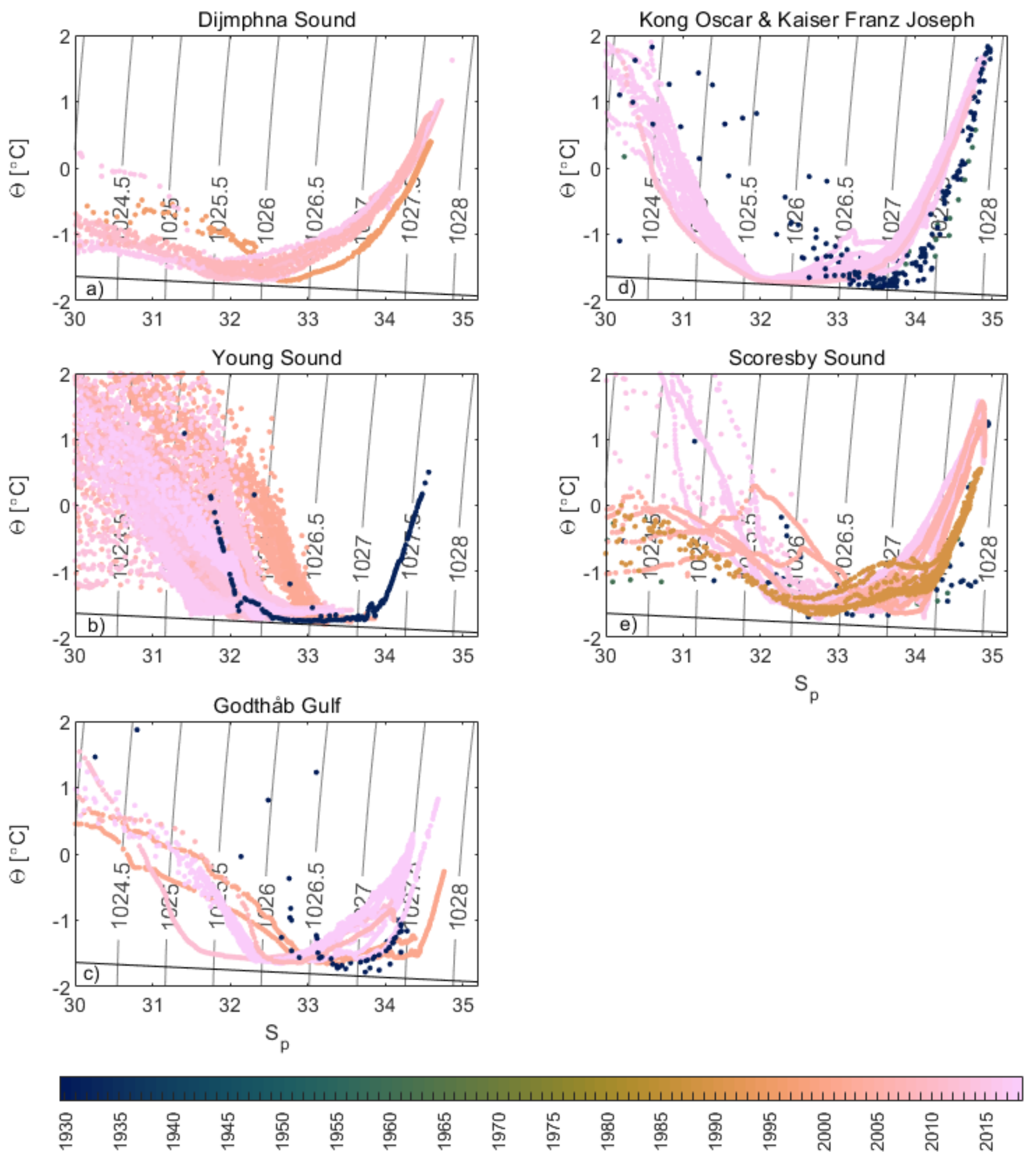

Fig. S3. Temperature-salinity (T/S) diagrams of all available fjord data during July/August/September. a) Dijmphna Sound, b) Young Sound, c) Godthåb Gulf, d) Kong Oscar & Kaiser Franz Joesph Fjord and e) Scoresby Sound. Measurements are colored by year of sampling. Mean T/S diagrams are provided on Fig. 2 in the main text.

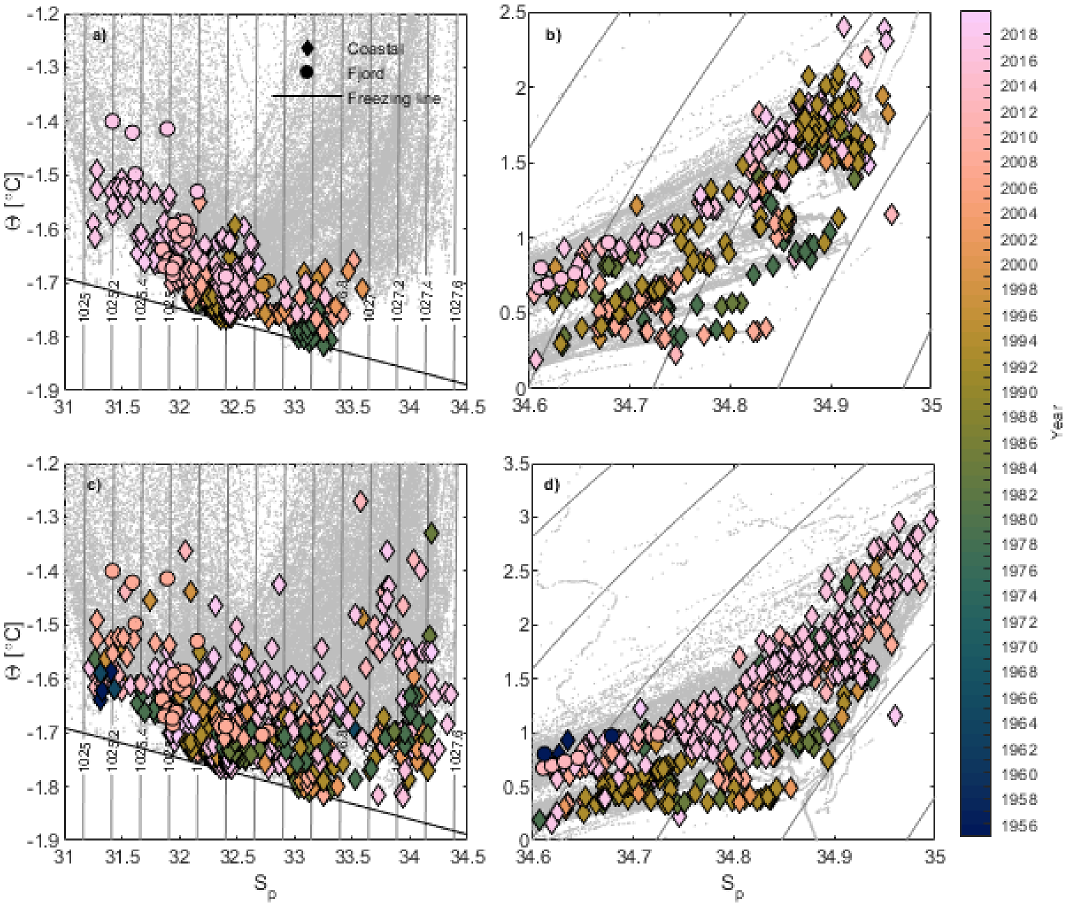

Fig. S4. Temperature-salinity (T/S) diagrams showing core Polar Water (panels a & c) and Atlantic Water (panels b & d) properties during July/August/September for the region 81.3°N-74.71°N. Fjord (circles) and shelf (diamonds) stations are superimposed. Time is given by color. Grey dots represent entire profiles. In the case of coarse vertical resolution these have been interpolated using the GSW function "gsw_SA_CT_interp" designed to respect the shape of the profile in T/S space. This is done solely for visualisation purposes. Panels a & b are based on data from within the near-shore shelf region defined by a polygon extending 100 km seaward from the shoreline unless the shelf is narrower, in which case the 500 m isobaths was used instead (see Fig. 1 in the main text). Panels c & d are based on shelf data west of the 500 m isobath. Note, increased scatter in Polar Water properties, and the appearance of warmer and more saline Atlantic Water in comparison to panels a & b.

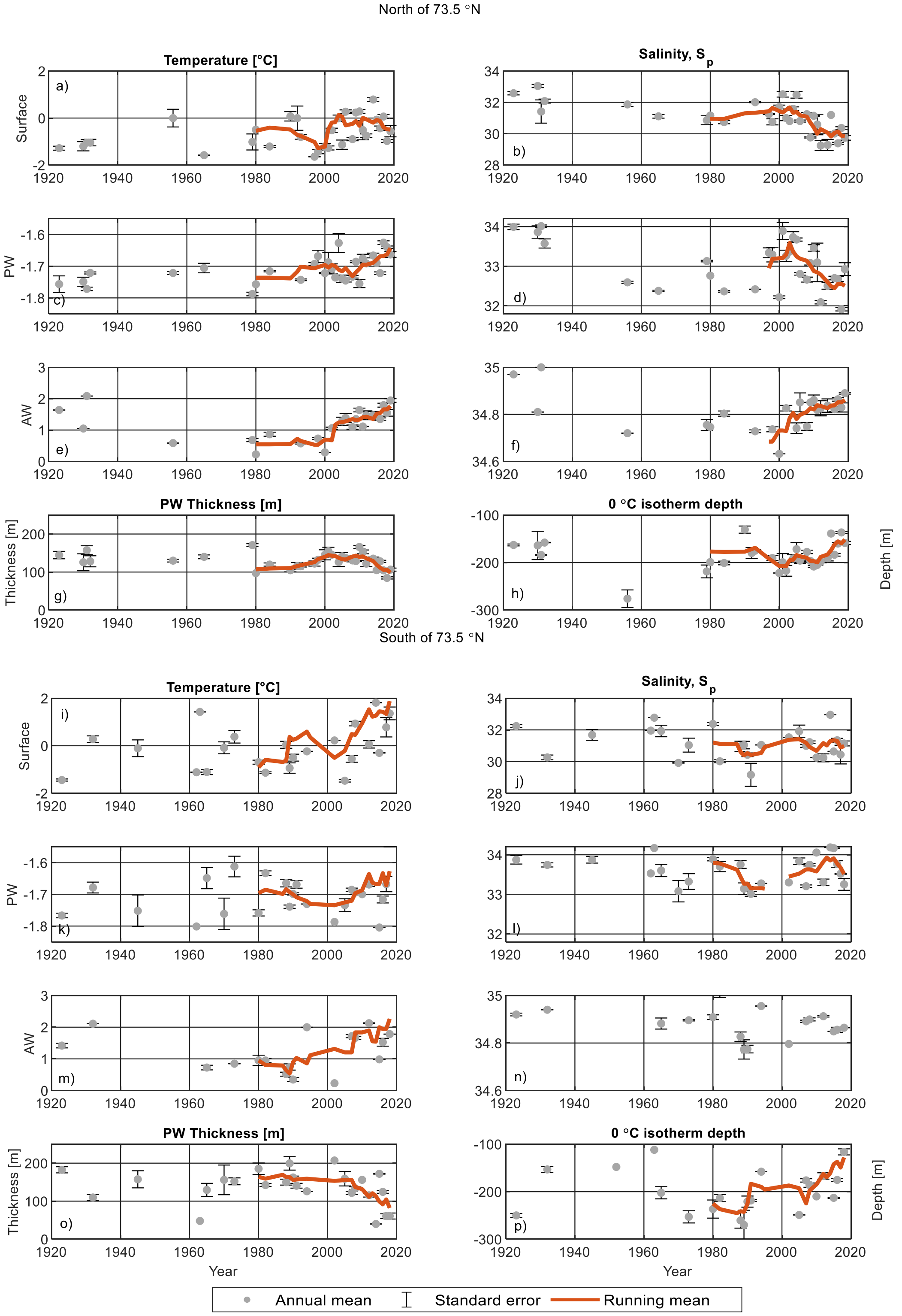

Fig. S5. Time series of summer (July/August/September) shelf water properties north (panels a-h) and south (panels i-p) of 73.5 °N. Surface water temperature (a & i) and salinity (b & j) defined as the mean of the 0-20 m depth interval, Polar Water (PW) core temperature (c & k), salinity (d & l) and layer thickness (g & o). Atlantic Water (AW) core temperature (e & m), salinity (f & n) and depth of the upper boundary of AW (h & p). Orange line shows the five-year running means and error bars are indicate the standard error.

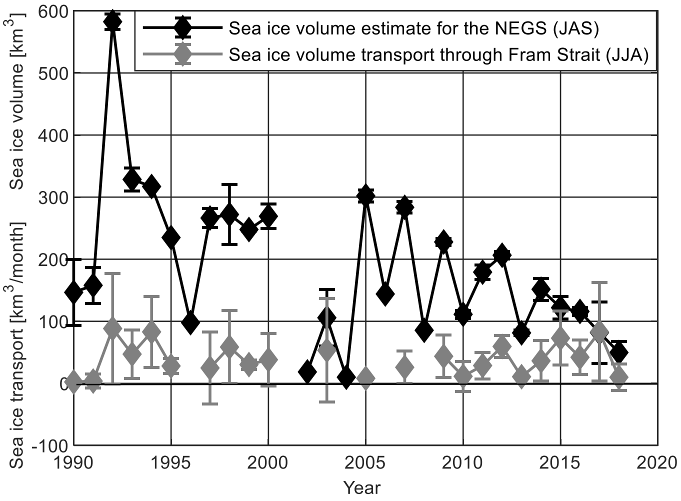

Fig. S6. Summer time sea-ice volume and transport. July-August-September (JAS) mean sea ice volume estimated over the Northeast Greenland Shelf (NEGS) and June-July-August (JJA) mean sea ice volume transport through the western Fram Strait. Error bars indicate one standard deviation.

Table S2. Observations complied for the current study.

Expedition/monitoring programme	Time	Month	Number of stations	Data source/reference
Various	1923, 1925-1926, 1929-1933, 1935-1936, 1938-1939, 1945, 1947-1952, 1954-1971, 1973-2019	Jan, Feb, Mar, Jul, Aug, Sep, Oct, Nov	344	ICES https://www.ices.dk/
Various	1929-1933, 1935-1936, 1938-1941, 1947-1952, 1954-1955, 1973, 1979-2019	Jan, Feb, Mar, Apr, May, Jun, Jul, Aug, Sep, Oct, Nov, Dec	1030	WOD https://www.ncei.noaa.gov/products/world-ocean-database
Various	1990-2019	Jul, Aug, Sep, Oct, Nov	1000	GTSPP https://www.ncei.noaa.gov/products/global-temperature-and-salinity-profile-programme
Norwegian expeditions to East Greenland (VESLEKARI and POLARBJØRN)	1930-1932	Jul, Aug	42	1
ARK-VII/3 (POLARSTERN)	1990	Jun, Jul, Aug, Sep	9	2
ARK-IX/3 (POLARSTERN)	1993	Jun, Jul, Aug	169	3
Nioghalvfjerdsfjorden glacier project	1997-1998	Jul, Aug	9	4
ARK-XIV/2 (POLARSTERN)	1998	Aug, Sep, Oct	24	5
ARK-XVII/1 (POLARSTERN)	2001	Jun, Jul	16	6
ARK XVIII/1a (POLARSTERN)	2002	Jul	7	7
ARK-XVIII/1b (POLARSTERN)	2002	Jul, Aug	24	8
ARK-XXIII/1 (POLARSTERN)	2008	Jun	28	9
ARK-XXIII/2 (POLARSTERN)	2008	Jul, Aug	70	10
ARCTIC SUNRISE	2009	Sep	27	11
ARK-XXIX/2.1 (POLARSTERN)	2015	Jul	2	12
MSM56 (MARIA S. MERIAN)	2016	Jul	10	13
PS100 (POLARSTERN)	2016	Jul, Aug, Sep	22	14
PS109 (POLARSTERN)	2017	Sep, Oct	127	15
Oceans Melting Greenland	2016-2019	Aug, Sep, Oct	130	NASA https://omg.jpl.nasa.gov/portal
TUNU expeditions	2002-2003, 2005, 2007, 2010, 2013, 2015	Aug, Sep, Oct	28	Christiansen pers coms.
Greenland Ecosystem Monitoring Programme	2003-2019	Jul, Aug, Sep	861	GEM https://data.g-e-m.dk/ Sejr & Winding pers coms.
Fram Strait	2012, 2015-2016	Aug, Sep	150	Dodd, de Steur, Pavlov and Kowalczuk pers coms.
NAACOS expedition (DANA)	2012	Aug	10	Stedmon pers coms.
NEG Ecosystem Assessment (DANA)	2017	Aug, Sep	83	Friis Møller pers coms.
East Greenland Fjords (LAKO)	2018	Aug	25	Sejr pers coms.

Supplementary References

1. Jakhelln, A. Oceanographic investigations in East Greenland waters in the summers of 1930-1932. *Skrifter om Svalbard og Ishavet* **67**, 1–79 (1936).
2. Cofaigh, C. Ó., Dowdeswell, J. A. & Grobe, H. Data from "Temperature and salinity from memory-CTD during POLARSTERN cruise ARK-VII/3". PANGAEA. Available at: https://doi.org/10.1594/PANGAEA.205502 (1996).
3. Budéus, G. & Schneider, W. Data from "Physical oceanography during POLARSTERN cruise ARK-IX/3. Bremerhaven, PANGAEA". Available at: https://doi.org/10.1594/PANGAEA.752257 (2010).
4. Schaffer, J. & Mayer, C. Data from "Physical oceanography during NE-Greenland expeditions 1997/98. PANGAEA". Available at: https://doi.org/10.1594/PANGAEA.891386 (2018).
5. Schauer, U. & Budéus, G. Data from "Physical oceanography during POLARSTERN cruise ARK-XIV/2. Bremerhaven, PANGAEA". Available at: https://doi.org/10.1594/PANGAEA.742655 (2010).
6. Budéus, G. Data from "Physical oceanography during POLARSTERN cruise ARK-XVII/1. Bremerhaven, PANGAEA". Available at: https://doi.org/10.1594/PANGAEA.742619 (2010).
7. Ronski, S. & Budéus, G. Data from "Physical oceanography during POLARSTERN cruise ARK-XVIII/1a. PANGAEA". Available at: https://doi.org/10.1594/PANGAEA.753139 (2010).
8. Schauer, U. & Rohardt, G. Data from "Physical oceanography during POLARSTERN cruise ARK-XVIII/1b. Bremerhaven, PANGAEA". Available at: https://doi.org/10.1594/PANGAEA.742620 (2010).
9. Budéus, G. & Wisotzki, A. Data from "Physical oceanography during POLARSTERN cruise ARK-XXIII/1. Bremerhaven, PANGAEA". Available at: https://doi.org/10.1594/PANGAEA.772614 (2011).
10. Beszczynska-Möller, A. & Wisotzki, A. Data from "Physical oceanography during POLARSTERN cruise ARK-XXIII/2. Bremerhaven, PANGAEA". Available at: https://doi.org/10.1594/PANGAEA.733424 (2010).
11. Wilson, N. J. & Straneo, F. Water exchange between the continental shelf and the cavity beneath Nioghalvfjerdsbræ (79 North Glacier). *Geophysical Research Letters* **42**, 7648–7654 (2015).

12. Von Appen, W.-J., Stein, R. & Wisotzki, A. Data from “Physical oceanography during POLARSTERN cruise PS93.1 (ARK-XXIX/2.1). Bremerhaven, PANGAEA”. Available at: https://doi.org/10.1594/PANGAEA.863063 (2016).
13. Friedrichs, A., Schwalfenberg, K., Koch, B. P. & Zielinski, O. Data from “Physical oceanography during MARIA S. MERIAN cruise MSM56 (MECAF). Germany, PANGAEA”. Available at: https://doi.org/10.1594/PANGAEA.871015 (2017).
14. Kanzow, T. *et al*., Data from ”Physical oceanography measured with CTD/Large volume Watersampler-system during POLARSTERN cruise PS100 (ARK- XXX/2). Bremerhaven, PANGAEA”. Available at: https://doi.org/10.1594/PANGAEA.871025 (2017).
15. Kanzow, T., Schaffer, J. & Rohardt, G. Data from “Physical oceanography during POLARSTERN cruise PS109 (ARK-XXXI/4). Bremerhaven, PANGAEA”. Available at: https://doi.org/10.1594/PANGAEA.885358 (2018).
